# Prevalence, Risk Factors, and Management of Metabolic Acidosis in Chronic Kidney Disease Patients: A Multicenter Retrospective Study in Malaysia

**DOI:** 10.7759/cureus.56314

**Published:** 2024-03-17

**Authors:** Jaime Yoke May Chan, Farida Islahudin, Nurul Ain Mohd Tahir, Mohd Makmor-Bakry, Clare Hui Hong Tan

**Affiliations:** 1 Center for Quality Management of Medicines, Faculty of Pharmacy, Universiti Kebangsaan Malaysia, Kuala Lumpur, MYS; 2 Faculty of Pharmacy, Universitas Airlangga, Surabaya, IDN; 3 Department of Nephrology, Hospital Umum Sarawak, Kuching, MYS

**Keywords:** sodium bicarbonate, serum bicarbonate, metabolic acidosis, chronic kidney disease (ckd), alkali therapy

## Abstract

Background

Metabolic acidosis in chronic kidney disease (CKD) patients has lately gained attention due to the growing evidence of its treatment benefits. This study aims to provide baseline data on the prevalence, risk factors, and current management of metabolic acidosis among the pre-dialysis adult Malaysian CKD population.

Methodology

This multicenter cross-sectional retrospective study involved pre-dialysis CKD patients above 18 years old on regular nephrology clinic follow-up at three Malaysian government hospitals with nephrology subspecialty. Demographic data, clinical information, laboratory data, and a list of concomitant medications were collected. Factors associated with the occurrence of metabolic acidosis were identified via multiple logistic regression.

Results

Six hundred and fifty-seven CKD patients were screened for this study, in which only 39.4% (n=259) had available bicarbonate levels. From this, a total of 86.1% (n=223) had metabolic acidosis. Higher estimated glomerular filtration rate (odds ratio (OR) 0.96, 95% confidence interval (CI) 0.93-1.00, p=0.043) and those with cardiovascular disease (OR 0.33, 95% CI 0.15-0.73; p=0.007) were significantly associated with lower odds of metabolic acidosis. There were 43.0% (n=96) on alkali therapy with sodium bicarbonate solution being the most common (n=91, 94.8%). Among those receiving alkali therapy, only 19.8% (n=19) achieved bicarbonate levels of ≥ 22 mEq/L.

Conclusion

Our study showed that metabolic acidosis was highly prevalent, although few achieved target levels despite supplementation, supporting the need for focused management of metabolic acidosis in the CKD population.

## Introduction

Metabolic acidosis, a known but often neglected complication of chronic kidney disease (CKD) has been gaining more attention in recent years [1-5]. The prevalence of metabolic acidosis among CKD patients varies from 6% to 80% depending on the CKD stages and the mode of renal replacement therapy, with Malaysia reporting a prevalence of 6.2% among its CKD population [6-9]. Metabolic acidosis in CKD patients happens when there is an imbalance between the kidney’s acid-excretory capacity and the daily acid load derived from dietary intake and protein metabolism [10]. Metabolic acidosis is defined as CKD patients having a serum bicarbonate of < 22 mEq/L [11]. As CKD progresses, metabolic acidosis becomes more apparent, prompting closer monitoring in later stages of CKD patients [7,8,12]. The monitoring for metabolic acidosis not only allows prompt diagnosis but also ensures proper correction. 

Appropriate management of metabolic acidosis contributes to minimizing CKD complications, which in turn is one of the major components in the CKD treatment plan. The potential benefits of correcting metabolic acidosis in CKD include slowing CKD progression, nutritional benefits, and better growth for children [2,13,14]. Alkali therapy has been advocated for the treatment of metabolic acidosis in CKD patients, with pharmacological management focusing on the oral administration of alkali salts [12]. Alkali therapy is generally well tolerated with minor gastrointestinal side effects due to its reaction in the stomach resulting in the production of carbon dioxide [15]. Previous studies have shown that treatment for metabolic acidosis with alkali therapy has not significantly increased the risk of worsening edema or hypertension in CKD patients [2,3,15,16]. However, good quality evidence is still lacking with regard to the benefits and risks of correcting metabolic acidosis in the CKD population.

The most common alkali therapy recommended is oral sodium bicarbonate. Oral sodium citrate and oral potassium citrate are other options available [5,10]. Formulations of oral sodium bicarbonate may differ from one institution to another and include tablet, capsule, or powder form [15]. Powder is usually dissolved in water prior to administration [10]. There are suggestions that the initiation of sodium bicarbonate supplementation be considered when serum bicarbonate is less than 22 mEq/L among CKD patients unless contraindicated [11,17]. However, there is still a lack of clear guidelines regarding the doses of bicarbonate supplementation, the frequency of bicarbonate level monitoring, and the optimal serum bicarbonate target level. Current recommendations for doses and target bicarbonate levels vary and are based on different studies [2,5,17]. Bicarbonate doses range from 1.8 g/day to 3.8 g/day administered two or three times a day [3,4,12,14], with a targeted bicarbonate range proposed between 24 to 26 mEq/L [5,10]. Regular monitoring of bicarbonate levels is important while on oral alkali supplementation as excessive doses may lead to metabolic alkalosis which can be associated with poor outcomes such as increased cardiovascular risk and vascular calcification [18].

In Malaysia, bicarbonate level monitoring may not be routine or readily available and there is a paucity of information regarding the prevalence and treatment for metabolic acidosis in the Malaysian CKD population. Therefore, this study aimed to determine the prevalence of metabolic acidosis, its potential risk factors, and current management among the pre-dialysis adult CKD population in Malaysia.

## Materials and methods

Study design

This was a multicenter cross-sectional retrospective study conducted in three government hospitals in Malaysia with Nephrology subspeciality. The inclusion criteria were adult patients >18 years, on regular Nephrology Clinic follow-up between October 1, 2021 and December 31, 2021, dialysis naive, with at least one laboratory reading during the study period. Patients with incomplete medical records and those with renal tubular acidosis were excluded. For those with more than one laboratory reading during the study period, only data from the first visit were analyzed. The study was performed in accordance with STROBE guidelines.

Ethics approval

The study was approved by the Ministry of Health (MOH) Medical Research and Ethics Committee (NMRR ID-22-00075-X9G) and the Universiti Kebangsaan Malaysia Research Ethic Committee (JEP-2022-651). The study was conducted in line with the ethical standards specified in the 1964 Declaration of Helsinki and its later amendments or comparable ethical standards. As the study was retrospective and non-interventional in nature, a consent waiver was granted.

Sample size

The sample size required [19], taking into account a confidence level of 95% precision of 5%, and an overall prevalence of metabolic acidosis of 15% [20], was a minimum of 196 CKD patients with metabolic acidosis. The sample size was deliberately increased to 223 for sub-analysis of data.

Definitions

The estimated Glomerular Filtration Rate (eGFR) was calculated using the Chronic Kidney Disease Epidemiology Collaboration (CKD-EPI) equation [21]. Serum creatinine was measured based on the Jaffe method in each respective hospital [22]. CKD classification for kidney damage was based on the eGFR levels where Stage 1 CKD is normal or high eGFR, with eGFR of 90 mL/min/1.73m^2^ or more; CKD Stage 2 is mildly decreased eGFR of 60 to 89 mL/min/1.73m^2^; CKD Stage 3a is mild to moderately decreased eGFR of 45 to 59 mL/min/1.73m^2^; CKD Stage 3b is moderate to severe eGFR of 30 to 44 mL/min/1.73m^2^; CKD Stage 4 is severely decreased eGFR of 15 to 29 mL/min/1.73m^2^ and CKD Stage 5 is eGFR of less than 15 mL/min/1.73m^2 ^[11]. Hyperkaliemia was defined as serum potassium of more than 5.0 mmol/L [20], while metabolic acidosis was defined as serum bicarbonate of less than 22 mEq/L [11].

Bicarbonate levels were measured either using serum or venous blood gas. Serum bicarbonate level was measured via the phosphoenolpyruvate carboxylase method on the Roche Diagnostic Cobas c702 module system, whilst venous blood gas was analyzed and calculated on the Roche Diagnostic Cobas b221 module system or the GEM Premier 3500 system.

Data collection

Data collection was carried out via convenience sampling. A standardized data collection form was used to collect data. The data collection form was validated through a pilot study to ensure that data could be retrieved from the medical records. The data collection form was divided into three sections: demographic and clinical information, laboratory data, and medications, if any.

Patient’s age, gender, ethnic group, co-morbidities, CKD stage, etiology of CKD, and laboratory data like serum creatinine, potassium, and bicarbonate levels were obtained from the patient’s Nephrology clinic cards (either hard copy or electronic version-based on the respective hospital). The laboratory tests were generally done two weeks prior to the scheduled clinic visit. All medications prescribed including information on alkali therapy prior to the current visit were recorded. Medication information was elicited from the patient’s Nephrology clinic cards or the Pharmacy Information System (PhIS). 

Statistical analysis

Statistical analysis was performed via the IBM Statistical Product and Service Solutions (SPSS) software (version 27; IBM SPSS Statistics for Windows, IBM Corp., Armonk, NY). Data were presented descriptively with frequency and percentage (%) for categorical variables whilst means, standard deviation (SD), median, and interquartile range (IQR) were for continuous variables. Comparison between groups for categorical variables was analyzed using the chi-square test or Fisher’s exact test whereas the independent t-test or Mann-Whitney U non-parametric test was used to compare the means of selected continuous variables. Univariate logistic regression analysis was performed on clinical and laboratory parameters comparing patients with and without metabolic acidosis. The factors associated with metabolic acidosis were assessed via univariate and multiple logistic regression analysis. Variables with p<0.25 or clinically meaningful in the univariate logistic analysis were included in the multiple logistic regression, followed by an examination of multicollinearity and correlation between the factors. Adjusted odd ratios (OR) with 95% confidence intervals (CIs) were reported. P-values of < 0.05 were considered statistically significant.

## Results

**Figure 1:**
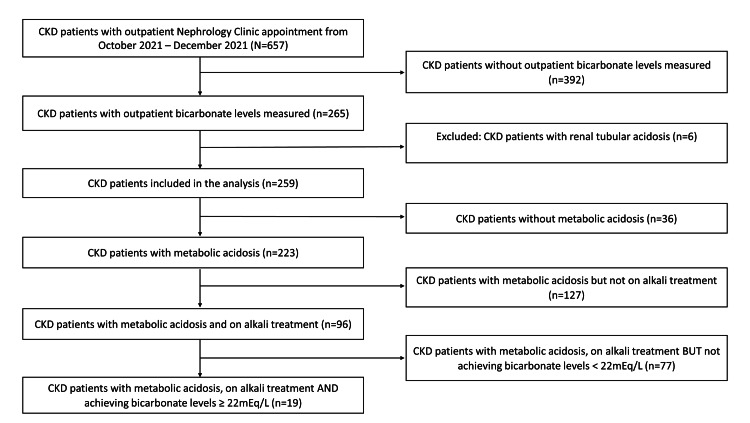
Study population selection flowchart The total number of chronic kidney disease (CKD) patients screened, N=657, but those with available bicarbonate levels for analysis, n=259 (six was excluded). Total number of CKD patients diagnosed with metabolic acidosis, n=223. Those currently on alkali treatment, n=96, but the number of CKD patients who achieved bicarbonate level ≥ 22mEq/L while on alkali treatment, n = 19.

Six hundred and fifty-seven CKD patients were screened for this study but only 39.4% (n=259) had available bicarbonate levels that were included in the analysis (Figure 1). From this, a total of 86.1% (n=223) had metabolic acidosis. The bicarbonate levels were either measured via serum (n=176, 68.0%) or venous blood gas (n=83, 32.0%).

The median (IQR) age of CKD patients in this study was 62 (19) years, with 50.2% (n=130) female and 44.8% (n=116) Malays. The majority had stage five pre-dialysis CKD, that is eGFR less than 15 mL/min/1.73m^2 ^(n=165, 63.7%) and the underlying cause of CKD was mainly diabetes mellitus (n=152, 58.7%) and hypertension (n=36, 13.9%). The median (IQR) number of co-morbidities was 3 (1) with hypertension being the most common co-morbidity seen (n=217, 83.8%). More than half of the CKD patients in this study were prescribed eight or more types of medications (n=136, 52.5%) (Table 1). 

**Table 1:** Demographic, medication, and clinical characteristics of the study population (n=259) ^a^Frequency, n (%), Chi-square test; ^b^Mean (standard deviation, SD), Independent t-test; ^c^Median (interquartile range, IQR), Mann-Whitney U non-parametric test; ^d^Indian ethnicity includes Punjabi (n=1); ^e^Others ethnicity include Iban (n=37) and Bidayuh (n=20); ^f^Number of medications was categorized using median split [23]; ^g^Others underlying cause of CKD include systemic lupus erythematosus (n=4), other renal causes (n=26), unknown causes (n=5), and uncertain (n=17); ^h^Bicarbonate levels expressed in mEq/L and mmol/L are equivalent; ^i^p <0.05 is statistically significant

Characteristics^a, b, c^	Total bicarbonate levels (n=259)	Metabolic acidosis (n=223)	No metabolic acidosis (n=36)	P-value^i^
Age				0.956
< 65 years (n, %)	150 (57.9)	129 (86.0)	21 (14.0)	
≥ 65 years (n, %)	109 (42.1)	94 (86.2)	15 (13.8)	
Gender				0.701
Male (n, %)	129 (49.8)	110 (85.3)	19 (14.7)	
Female (n, %)	130 (50.2)	113 (86.9)	17 (13.1)	
Ethnicity				0.342
Malay (n, %)	116 (44.8)	96 (82.8)	20 (17.2)	
Chinese (n, %)	72 (27.8)	65 (90.3)	7 (9.7)	
Indian^d ^(n, %)	14 (5.4)	11 (78.6)	3 (21.4)	
Others^e ^(n, %)	57 (22.0)	51 (89.5)	6 (10.5)	
Number of medications^f^				0.078
Less than 8 (n, %)	123 (47.5)	101 (82.1)	22 (17.9)	
8 or more (n, %)	136 (52.5)	122 (89.7)	14 (10.3)	
On Alkali therapy (n, %)	96 (37.1)	96 (100.0)	NA	-
eGFR (mL/min/1.73m^2^) (median, IQR)	12.64 (9.68)	12.16 (9.06)	16.35 (12.62)	0.002
Underlying cause of CKD				0.656
Diabetes mellitus (n, %)	152 (58.7)	134 (88.2)	18 (11.8)	
Hypertension (n, %)	36 (13.9)	29 (80.6)	7 (19.4)	
Glomerulonephritis (n, %)	19 (7.3)	16 (84.2)	3 (15.8)	
Others^g ^(n, %)	52 (20.1)	44 (84.6)	8 (15.4)	
Number of Co-Morbidities(median, IQR)	3 (1)	2 (1)	3 (1)	0.302
Type of Co-Morbidities				
Diabetes Mellitus (n, %)	172 (66.4)	150 (87.2)	22 (12.8)	0.468
Hypertension (n, %)	217 (83.8)	187 (86.2)	30 (13.8)	0.937
Cardiovascular disease (n, %)	55 (21.2)	40 (72.7)	15 (27.3)	0.001
Dyslipidaemia (n, %)	36 (13.9)	31 (86.1)	5 (13.9)	0.998
Gout (n, %)	42 (16.2)	37 (88.1)	5 (11.9)	0.683
Cerebrovascular diseases (n, %)	19 (7.3)	15 (78.9)	4 (21.1)	0.349
Blood pressure > 130/80 mmHg (n, %)	185 (71.4)	160 (86.4)	25 (13.5)	0.776
Serum potassium>5.0 mmol/L (hyperkalaemia) (n, %)	76 (29.3)	68 (89.5)	8 (10.5)	0.312
Bicarbonate levels (mEq/L^h^) (mean ± SD)	18.99 ± 3.75	18.17 ± 3.30	24.07 ± 1.84	0.001

Prevalence of metabolic acidosis

The prevalence of metabolic acidosis in this study was 86.1% (n=223), of which 96 patients (43.0%) were already on alkali therapy whilst the remaining 127 patients (57.0%) were not on treatment. Further analysis showed that the mean bicarbonate level in the metabolic acidosis group not on treatment was 17.03 (SD 2.99) mEq/L versus 19.69 (SD 3.09) mEq/L in the group already on alkali therapy and this was statistically significant (p < 0.001). Although metabolic acidosis was frequently reported in CKD patients with diabetes mellitus (n=134, 88.2%), there was no significant difference in the prevalence of metabolic acidosis among the various primary causes of CKD in this study. The prevalence of metabolic acidosis among patients with serum potassium more than 5.0 mmol/L was 89.5% (n=68).

Risk factors associated with metabolic acidosis

The univariate logistic regression analysis included variables which were eGFR, the presence of cardiovascular disease as a comorbidity, and a number of medications. The multiple logistic regression analysis in Table 2 showed that higher eGFR and the presence of cardiovascular disease were significantly associated with lower odds of metabolic acidosis. For every 1 mL/min/1.73m^2^ increase in eGFR, the odds of having metabolic acidosis were reduced by 4% (OR 0.96, 95% CI 0.93-1.00, p=0.043) whilst the presence of cardiovascular disease reduced the odds of having metabolic acidosis by 67% (OR 0.33, 95% CI 0.15-0.73; p=0.007). The model demonstrated an acceptable fit with the Hosmer and Lemeshow test reporting no significance (ꭕ^2^(8) = 5.46, p=0.707), and interactions and multicollinearity between both variables were checked and not found. The model was able to accurately discriminate 68.1% (95% CI 0.58-0.78, p=0.001) of the cases.

**Table 2:** Univariate and multiple binary logistic regression of factors associated with metabolic acidosis (n=163) ^a^eGFR: estimated glomerular filtration rate; ^b^OR: odds ratio; ^c^CI: confidence interval; ^d^p <0.05 is statistically significant

Variable (Reference)	Univariate Logistic Regression		
	OR^b^	95% CI^c^	P-value^d^
Age ≥ 65 years (<65 years)	0.939	0.44-1.99	0.871
Female (male)	1.209	0.58-2.54	0.615
Ethnicity (Malay)	1.00		0.065
Chinese	2.262	0.87-5.91	0.096
Indian	0.417	0.08-2.24	0.308
Others	2.639	0.96-722	0.059
eGFR^a^ (mL/min/1.73m^2^)	0.964	0.93-1.00	0.041^d^
Underlying cause of chronic kidney disease (Others)	1.00		0.424
Diabetes mellitus	1.672	0.64-4.38	0.295
Hypertension	0.762	0.23-2.58	0.662
Glomerulonephritis	1.651	0.37-7.37	0.511
Type of co-morbidities			
Diabetes mellitus (none)	1.160	0.54-2.49	0.703
Hypertension (none)	1.010	0.37-2.73	0.985
Cardiovascular disease (none)	0.326	0.15-0.72	0.006
Dyslipidemia (none)	0.588	0.19-1.82	0.356
Gout (none)	1.091	0.38-3.16	0.873
Cerebrovascular disease (none)	0.610	0.18-2.11	0.435
Blood pressure > 130/80mmHg (≤ 130/80mmHg)	1.112	0.50-2.49	0.796
Serum potassium > 5.0 mmol/L (≤5.0 mmol/L)	1.280	0.53-3.08	0.582
Number of medications >8 medications (<8 medications)	1.755	0.83-3.73	0.144
Variable (Reference)	Multiple Logistic Regression		
	Adjusted OR^b^	95% CI^c^	P-value^d^
eGFR^c^ (mL/min/1.73m^2^)	0.964	0.93-1.00	0.043
Cardiovascular disease (none)	0.326	0.15-0.73	0.007

Management of metabolic acidosis

The proportion of CKD patients with metabolic acidosis on alkali therapy was 43.0% (n=96). The types of alkali therapy used were oral sodium bicarbonate solution (n=91, 94.8%), oral sodium bicarbonate tablet (n=1, 1.04%), sodium citrate/citrate acid (Shohl’s) solution (n=1, 1.04%), and Ural® solution (n=2, 2.08%). Only 19.8% (n=19) of those on alkali therapy achieved the targeted bicarbonate levels of ≥ 22 mEq/L (Table 3). Overall, the dose of sodium bicarbonate prescribed for this study population ranged from 0.5 to 15 g/day, with a median (IQR) dose of 2 (2) g/day. Further analysis demonstrated that the only significant association for the achievement of targeted bicarbonate levels was the daily dose of oral sodium bicarbonate taken (median (IQR) dose 3 (2.25) g/day, *z* statistics -2.224, p=0.026). No significant association was shown with other patient characteristics.

**Table 3:** Types of alkali therapy (n=96) ^a^Ural® (4 g) contains sodium bicarbonate (1.76 g), tartaric acid (890 mg), citric acid anhydrous (720 mg), sodium citrate anhydrous (630 mg), sodium saccharin and lemon flavor; ^b^Combination consists of sodium bicarbonate solution and Shohl's solution (n=1).

Characteristics	Total on alkali treatment (n=96)	Achieved bicarbonate target (n=19)	Did not achieve bicarbonate target (n=77)
Types of alkali therapy prescribed (n, %)			
Sodium bicarbonate solution	91 (94.8)	18 (19.8)	73 (80.2)
Sodium bicarbonate tablet	1 (1.04)	0 (0.0)	1 (100.0)
Sodium citrate/citrate acid solution (Shohl's)	1 (1.04)	0 (0.0)	1 (100.0)
Ural®^a^	2 (2.08)	1 (50.0)	1 (50.0)
Combination^b^	1 (1.04)	0 (0.0)	1 (100.0)

## Discussion

The current study showed that bicarbonate level monitoring was not routine for CKD patients in the outpatient settings in most government hospitals in Malaysia, similar to what was reported previously [10]. This is evident, as bicarbonate levels were only available in 39.4% of our study population, of which the majority demonstrated metabolic acidosis. This is a stark difference compared to studies from other countries where the baseline bicarbonate levels were available for more than 70% of their patients [8,24]. The diagnosis of metabolic acidosis in CKD is usually made when the assessment of bicarbonate concentration in venous plasma or venous blood is below 22mEq/L [12]. The recommended frequency for bicarbonate level monitoring in CKD patients is every three months among those in stages 4 and 5 [25]. It may also be warranted for stage 3 CKD based on emerging evidence of the benefits of metabolic acidosis treatment in this kidney population [25,26]. Complications associated with metabolic acidosis includes increased risk of bone fractures due to the reduction in bone mineral density, increase skeletal muscle tissue loss, insulin resistance and impaired parathyroid hormone balance [3,12,27]. Therefore, the presence of metabolic acidosis complicates the management of CKD [28]. The lack of monitoring makes management of metabolic acidosis challenging as monitoring is needed to guide initiation of treatment, assess treatment response or adherence, adjust treatment dose and to avoid overtreatment with alkali therapy.

Among those with bicarbonate levels, the prevalence of metabolic acidosis was observed in approximately three-quarters of the study population. A French study analyzing 1,038 adults with stages 2 through 5 CKD not on dialysis reported the overall prevalence of metabolic acidosis of 15% and around 39% in the subset of 160 CKD patients with a measured GFR < 20 mL/min/1.73m^2^ [20]. Other work reports a prevalence of metabolic acidosis of 17.3% [8], and 24% depending on the CKD stage or the type of dialysis [7]. The prevalence of metabolic acidosis in Malaysia reported thus far was 6.2% among 204 kidney transplant recipients [9]. The prevalence in the current study was higher, possibly due to the fact that the majority of the study population were in CKD stage 4-5 as metabolic acidosis happens more frequently in those with more advanced stages of CKD [7,8,20]. Moreover, the study population was not on any type of renal replacement therapy yet. Other possible reasons may include dietary factors which was not explored in our study.

One factor associated with the occurrence of metabolic acidosis in our study was a reduction in eGFR. Similar findings were shown in previous studies. Results from the Third National Health and Nutrition Examination Survey (NHANES III) showed a significant association between acidosis and kidney clearance of less than 30 mL/min/1.73m^2^ and only a weak association for kidney clearance between 30 and 60 mL/min/1.73m^2 ^[26]. In another study, among 37,346 CKD patients, a higher eGFR was reported to be associated with lower odds of low serum bicarbonate levels [24]. In addition to this, analysis of the baseline data from the Chronic Renal Insufficiency Cohort (CRIC) study of 3,939 participants with an eGFR of 20 to 70 mL/min/1.73m^2^ also reported a strong relationship between lower eGFR and higher odds of low serum bicarbonate [8]. Kidneys play a key role in acid excretion and as the eGFR decreases, the kidneys are unable to excrete the acid load effectively resulting in a positive hydrogen ion balance and metabolic acidosis [10,28].

Our study also showed that CKD patients with concurrent cardiovascular disease was a significant factor in a lower risk of metabolic acidosis. This interesting observation was also reported by Navaneethan et. al. where the presence of congestive heart failure (CHF) was associated with lower odds of low serum bicarbonate levels [24]. CHF patients have been reported to have various acid-base disorders resulting from renal loss of hydrogen ions, movements of hydrogen ions into the cells, reduction in the effective circulating volume, hypoxemia and renal failure; with metabolic alkalosis being the most common acid-base disturbance. Apart from that, the use of diuretics in CHF patients have been noted to increase the chance of occurrence of metabolic alkalosis [29]. However, this association was not observed in the baseline data of the CRIC study [8], demonstrating the need for further work in this area.

Pharmacological therapy should only be considered for CKD patients with a confirmed diagnosis of metabolic acidosis. In this study, less than half of the CKD patients with metabolic acidosis was started on pharmacological therapy. Furthermore, for those on bicarbonate treatment, less than 20% achieved the target bicarbonate level. The only significant association with achieving target bicarbonate level was the dose of bicarbonate therapy. The doses of oral sodium bicarbonate used in the current study were between 0.5 and 15 g/day, with most patients on 2 g/day. Doses in previous studies varied between 1 and 6 g/day [2,5]. Interestingly, studies in the Asia region reported a slightly lower dose range as compared to the Western counterparts, with an average of 1.9 to 3 g/day [3,4,14], similar to doses used in our current study. Most patients in this study were on oral sodium bicarbonate powder reconstituted in solution form, which is currently the formulation available in the MOH facilities. However, this may be inconvenient and sodium bicarbonate powder reconstituted in water tastes slightly bitter and leaves an aftertaste in the mouth leading to potential nonadherence to therapy. This may be one of the reasons for our low percentage of patients achieving the bicarbonate target.

There were a few limitations to our study. The first being the small sample size of patients being monitored for metabolic acidosis and the involvement of only three MOH hospitals. Hence, the results may not be a representation of the whole Malaysian CKD population. For the diagnosis of metabolic acidosis, we only used one single bicarbonate value and accepted different assay methods because bicarbonate level monitoring in the outpatient clinic setting is not a common practice yet in our country. Although the evaluation of blood pH contributes to a more accurate acid-base diagnosis, blood gas analysis is not readily available in the outpatient clinical practice [30] and was not available in two of the MOH hospitals in this study. As this study was done retrospectively in 2022, the bicarbonate level of <22 mEq/L as indication for treatment initiation for metabolic acidosis was based on the most recent guidelines available in that year [11]. Another limitation of this study was that CKD patients with missing laboratory values during the study period were excluded, which could affect the overall prevalence. Patient’s diet and adherence to alkali therapy was also not assessed, which could contribute to the management of metabolic acidosis in some patients. Therefore, generalizability of the results should be done with caution.

## Conclusions

This study successfully highlighted that metabolic acidosis is highly prevalent among CKD patients and increases with more advanced stages of CKD. However, monitoring was infrequent, which led to low treatment rate and few corrected metabolic acidosis. The complexity of the management of metabolic acidosis would therefore require a multidisciplinary approach consisting of clinicians, nurses, pharmacists and dieticians. Further studies are needed to explore ways to promote bicarbonate monitoring, investigate the optimal doses of sodium bicarbonate therapy and ways to achieve target bicarbonate levels.

## References

[1] Whitlock RH, Ferguson TW, Komenda P (2023). Metabolic acidosis is undertreated and underdiagnosed: a retrospective cohort study. Nephrol Dial Transplant.

[2] Di Iorio BR, Bellasi A, Raphael KL (2019). Treatment of metabolic acidosis with sodium bicarbonate delays progression of chronic kidney disease: the UBI Study. J Nephrol.

[3] Dubey AK, Sahoo J, Vairappan B, Haridasan S, Parameswaran S, Priyamvada PS (2020). Correction of metabolic acidosis improves muscle mass and renal function in chronic kidney disease stages 3 and 4: a randomized controlled trial. Nephrol Dial Transplant.

[4] Kittiskulnam P, Srijaruneruang S, Chulakadabba A, Thokanit NS, Praditpornsilpa K, Tungsanga K, Eiam-Ong S (2020). Impact of serum bicarbonate levels on muscle mass and kidney function in pre-dialysis chronic kidney disease patients. Am J Nephrol.

[5] Melamed ML, Raphael KL (2021). Metabolic acidosis in CKD: a review of recent findings. Kidney Med.

[6] Kim HJ, Ryu H, Kang E (2021). Metabolic acidosis is an independent risk factor of renal progression in Korean chronic kidney disease patients: the KNOW-CKD study results. Front Med (Lausanne).

[7] Kuczera P, Ciaston-Mogilska D, Oslizlo B, Hycki A, Wiecek A, Adamczak M (2020). The prevalence of metabolic acidosis in patients with different stages of chronic kidney disease: single-centre study. Kidney Blood Press Res.

[8] Raphael KL, Zhang Y, Ying J, Greene T (2014). Prevalence of and risk factors for reduced serum bicarbonate in chronic kidney disease. Nephrology (Carlton).

[9] Lau WP, Ng KP, Ganapathy SS, Tah PC, Ismail R, Jalalonmuhali M, Lim SK (2022). Prevalence rate of proteinuria and metabolic acidosis among kidney transplant recipients in a tertiary teaching hospital and its relationship to dietary intake. Transplant Proc.

[10] Raphael KL (2019). Metabolic acidosis in CKD: Core Curriculum 2019. Am J Kidney Dis.

[11] (2013). KDIGO 2012 clinical practice guideline for the evaluation and management of chronic kidney disease. Kidney Int Suppl (2011).

[12] Adamczak M, Surma S (2021). Metabolic acidosis in patients with CKD: epidemiology, pathogenesis, and treatment. Kidney Dis (Basel).

[13] Brown DD, Carroll M, Ng DK (2022). Longitudinal associations between low serum bicarbonate and linear growth in children with CKD. Kidney360.

[14] Jeong J, Kwon SK, Kim HY (2014). Effect of bicarbonate supplementation on renal function and nutritional indices in predialysis advanced chronic kidney disease. Electrolyte Blood Press.

[15] Łoniewski I, Wesson DE (2014). Bicarbonate therapy for prevention of chronic kidney disease progression. Kidney Int.

[16] Beynon-Cobb B, Louca P, Hoorn EJ, Menni C, Padmanabhan S (2023). Effect of sodium bicarbonate on systolic blood pressure in CKD: a systematic review and meta-analysis. Clin J Am Soc Nephrol.

[17] Kraut JA, Madias NE (2016). Metabolic acidosis of CKD: an update. Am J Kidney Dis.

[18] Dobre M, Rahman M, Hostetter TH (2015). Current status of bicarbonate in CKD. J Am Soc Nephrol.

[19] Lemeshow S, Hosmer DW, Klar J, Lwanga SK (1990). Adequacy of sample size in health studies. https://scholar.google.com/scholar_lookup?journal=World+Health+Organization&title=Adequacy+of+sample+size+in+health+studies&author=S+Lemeshow&author=DW+Hosmer&author=J+Klar&author=SK+Lwanga&publication_year=1990&pages=1-4&.

[20] Moranne O, Froissart M, Rossert J (2009). Timing of onset of CKD-related metabolic complications. J Am Soc Nephrol.

[21] Teo BW, Xu H, Wang D (2011). GFR estimating equations in a multiethnic Asian population. Am J Kidney Dis.

[22] Delanghe JR, Speeckaert MM (2011). Creatinine determination according to Jaffe-what does it stand for?. NDT Plus.

[23] Iacobucci D, Posavac SS, Kardes FR, Schneider MJ, Popovich DL (2015). Toward a more nuanced understanding of the statistical properties of a median split. J Consum Psychol.

[24] Navaneethan SD, Schold JD, Arrigain S (2011). Serum bicarbonate and mortality in stage 3 and stage 4 chronic kidney disease. Clin J Am Soc Nephrol.

[25] Eknoyan G, Levin A, Levin NW (2003). Bone metabolism and disease in chronic kidney disease. Am J Kidney Dis.

[26] Clase CM, Kiberd BA, Garg AX (2007). Relationship between glomerular filtration rate and the prevalence of metabolic abnormalities: results from the Third National Health and Nutrition Examination Survey (NHANES III). Nephron Clin Pract.

[27] Kraut JA, Madias NE (2017). Adverse effects of the metabolic acidosis of chronic kidney disease. Adv Chronic Kidney Dis.

[28] Chen W, Levy DS, Abramowitz MK (2019). Acid base balance and progression of kidney disease. Semin Nephrol.

[29] Urso C, Brucculeri S, Caimi G (2015). Acid-base and electrolyte abnormalities in heart failure: pathophysiology and implications. Heart Fail Rev.

[30] Kraut JA, Raphael KL (2021). Assessment of acid-base status: beyond serum bicarbonate. Clin J Am Soc Nephrol.

